# The emerging paradigms of SETD family enzymes as epigenetic regulators of the immune response in inflammatory diseases

**DOI:** 10.3389/fimmu.2026.1725917

**Published:** 2026-01-23

**Authors:** Chunhui Liu, Lei Lin, Guoliang Yao, Yonggang Fan, Yongjun Guo

**Affiliations:** 1 The First Affiliated Hospital,and College of Clinical Medicine of Henan University of Science and Technology, Luoyang, China; 2 Henan Key Laboratory of Molecular Pathology, Zhengzhou, China; 3 Luoyang Central Hospital Affiliated to Zhengzhou University, Luoyang, China; 4 The First Affiliated Hospital of Henan University of Science and Technology, Luoyang, China; 5 Department of Molecular Pathology, The Affiliated Cancer Hospital of Zhengzhou University, Henan Cancer Hospital, Zhengzhou, China

**Keywords:** epigenetic therapy, histone methylation, immune epigenetics, inflammatory diseases, SETD

## Abstract

Family members of the SET domain family (SETD) of histone lysine methyltransferases (HKMTs) act as principal epigenetic regulators, modulating chromatin structure, transcription pathways, and immune responses. SETDs catalyze lysine methylation on histone and non-histone substrates, as well as non-histone proteins (e.g., p53, NF-κB). These biochemical modifications support gene activity requisite for directing immune cells, modulating cytokine cascades, and inflammatory responses. For SETD family members, systemic dysregulation has become the principal mechanistic fulcrum within the orchestration of major autoimmune and inflammatory syndromes, comprising rheumatoid arthritis (RA) and systemic lupus erythematosus (SLE), psoriasis, atherosclerosis and type 2 diabetes, and, to a lesser extent, multiple sclerosis (MS) and inflammatory bowel disease (IBD). SETD1A and SETD1B catalyze H3K4 methylation and regulate the chromatin states governing the proliferation of T-lymphocytes. SETD2 spatially regulates H3K36 trimethylation with the augmentation of DNA regulatory steps and cytokine signaling. SETD6 and SETD7, and other components, enhance the NF-κB signaling involving innate immune response and regulation of chromatin structure. Experimentally validated mutations transform transcript re-equilibration and catalysis of benign enzymes. These alterations disturb immune consistency and endorse predetermined inflammatory responses, and weaken self-tolerance. In the post-genomic era, integrated therapeutic approaches are emerging from potent SETD modulators, small inhibitors, epigenetic scissors, and multi-omics techniques. Overall, this review demonstrates the emerging domain of immuno-epigenetics, SETD enzymes, and the strategic value they could serve as therapeutic targets and biomarkers.

## Graphical abstract

**Figure:**
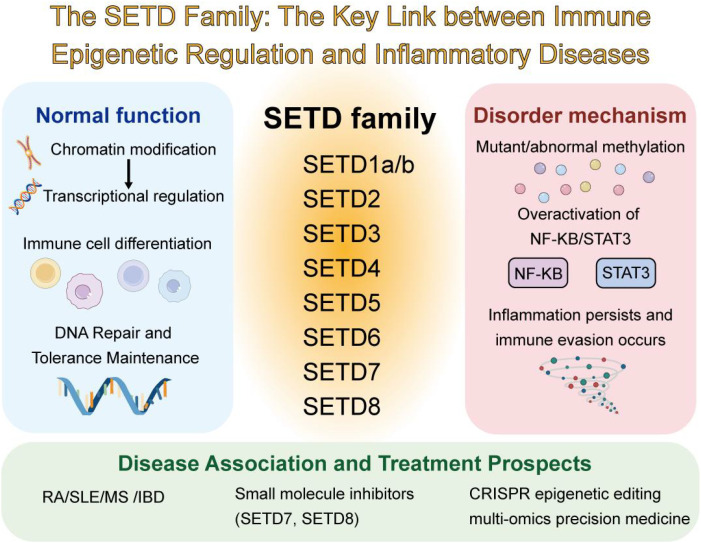

## 1 Introduction

The immune system must balance defending against pathogenic organisms with tolerance to the body’s own macromolecules, requiring the coordination of multi-step molecular and cellular interactions (1). When there is an imbalance, a whole range of constitutional and inflammatory autoimmune disorders could arise (2). Current reports indicate that, in addition to inherited genomic sequences, a multitude of epigenetic factors are critically important for the immune cell differentiation, lineage specification, and functional reprogramming processes (3, 4). Out of the many factors, the addition and removal of specific groups of certain chemicals to the histone proteins remain a layer of primary, central control since those factors balance how tightly chromatin is folded, and thus how many copies of a particular gene are accessible for transcription (5, 6). The different types of histone modifications include acetylation (HATs (p300/CBP, GCN5, PCAF), methylation (HMTs/SET domain enzymes, EZH2, SETDB1, DOT1L, ITGB3), phosphorylation (kinases (JAK2, Aurora B, MSK1, STE, AGC family), ubiquitylation (misfolded proteins, E3 ligases, RNF20/40, PRC1), SUMOylation (TET3), GlcNAcylation (fucosylated alpha-1-acidglycoprotein, alpha-2,6-sialylation), succinylation (Sirtuin5), lactylation p300, (METTL3)m6A), citrullination (GPX4), and crotonylation (p53), which are catalyzed by a specific set of enzymes (7–11). Over the past few decades, the methylation of histone (H) lysine (K), arginine, and histidine residues, a reversible modification catalysed by discrete families of methyltransferases, has been reported (12). Methylation of lysine by methyltransferases has been characterised as a conserved and decisive signalling axis that ultimately directs immune surveillance, tolerance, and the variable outcomes of inflammatory diseases (**Table 1**) (12, 13).

**Table 1:** Methylation patterns of histones.

Histone modification	Remarks
H3K4me1	Activates gene expression in the enhancer region (1, 13).
H3K4me2	Activation or repression of gene bodies (13)
H3K4me3	Repression of gene promoter region (13)
H3K9me2	Repression of transposable elements, satellite repeats, and gene bodies (14)
H3K9me3	Repression of transposable elements, telomeres, satellite repeats, and gene bodies (1, 14)
H3K27me3	Repression of enhancers and promoters, role in inflammation & tumor progression (1, 15)
H3K36me3	Activation of gene bodies (16)

### 1.1 Epigenetic directives and immune homeostasis

Epigenetic regulation refers to covalent modifications of chromatin that are stably inherited yet reversible and that do not involve alteration of the nucleotide sequence itself (17, 18). The primary mechanisms that contribute to this regulation include the methylation of cytosines in DNA, histones, and other proteins, as well as the activity of non-coding RNAs that scaffold or direct chromatin-remodelling complexes (19, 20). Within the immune system, epigenetic circuits calibrate transcriptional programs that control the differentiation of hematopoietic stem cells, the specification of helper T-cell subsets, and the functional reprogramming of tissue-resident macrophages and dendritic cells in response to diverse signals (2, 19). Epigenetic approaches provide strategies to maintain homeostasis in selective immune responses associated with host-pathogen interactions (4, 21). Deviations from epigenetic modifications may trigger hyperactive immune pathways, autoimmunity, and inflammatory tissue damage. Therefore, such modifications are crucial in the long-term preservation of immune homeostasis (22–24).

### 1.2 Histone lysine methylation as a key governing mechanism

**Figure 1:**
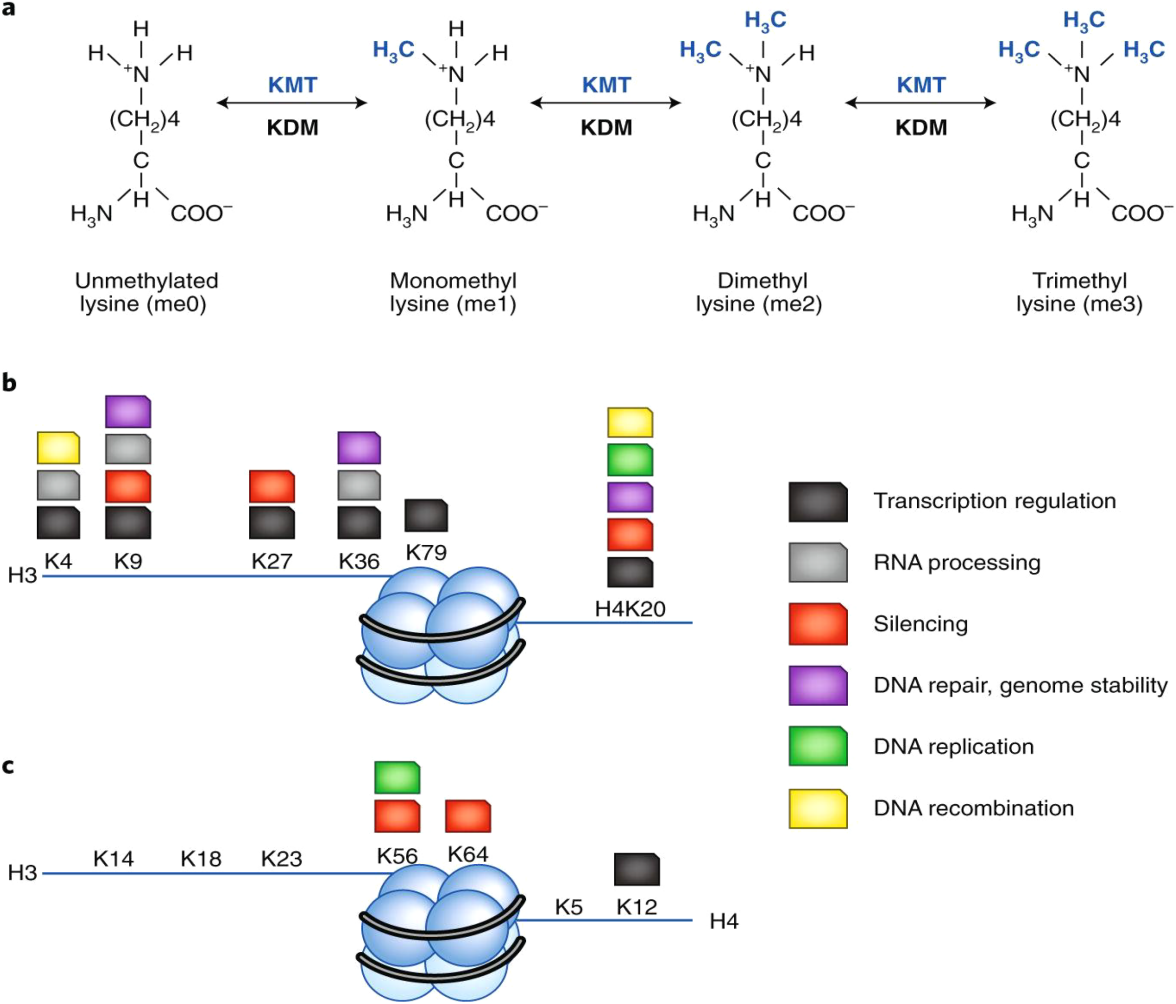
Principal sites of lysine methylation on mammalian histones and their impact on chromatin function. **(a)** Chemically modified lysine derivatives. Each residue exhibits mono-, di-, or trimethylation. **(b, c)** Histone H3 and H4 core self-markings. **(b)** Conventional lysine methylation; **(c)** alternative marks. Histidine methylation positions are highlighted on histones with indicated colors (3).

Lysine (K) methylation of histone (H) tails is a highly adaptable chromatin modification. It is involved in the regulation of eukaryotic gene expression by imposing finely graded and context-sensitive marks (**Figure 1**) (19). Gene expression is activated or repressed depending on the site and degree of lysine methylation; mono-, di- or trimethylation. For instance, methylation of H3K4, H3K79 and H4K20 is linked with the activation of transcription (13). Methyltransferases and demethylases, along with methyl-lysine binding effectors, play a crucial role in these modifications (13, 25). Trimethylation of histone H3 at lysine 4 (H3K4me3), for example, is a hallmark of active promoters, whereas trimethylation of H3 lysines 9 (H3K9me3) and 27 (H3K27me3) typically marks facultative and constitutive heterochromatin, respectively (14). Methylation of lysine 36 on histone 3 (H3K36) has been linked to chromatin disassembly, transcription and methylation of DNA (26). H3K36m1 modification has a limited impact on transcription, and H3K36me2 is found in regulatory portions of the gene. H3K36me3, by contrast, functions predominantly in the maintenance of elongating polymerase II (9, 27). These methylation patterns have been catalyzed by a family of enzymes known as nuclear receptor-binding SET domain (NSD) methyltransferases (28). H4K20me1 is augmented in regions of nascent replicated DNA, linking these methylation patterns to DNA repair and the maintenance of overall genomic architecture (16, 29). As a result of these modifications, the outcomes of transcription are reversible and persistent in responding to development, metabolism, and environmental stimuli (30). In the hematopoietic compartment, the marks deposited and maintained by the enzymes configure the cytokines, surface receptors, and specific transcriptional regulators for the cell line (29, 31). The persistence modifications are also related to the enzymes for the cytokines in homeostasis, homeorhetic responses and inflammation. The deviation of the modified histones may account for dysregulated mechanisms related to autoimmune disorders, chronic inflammation, and immune dysregulation associated with cancer (2, 32).

### 1.3 An overview of the SETD family of proteins

The SET domain-containing (SETD) family of methyltransferases comprises a heterogeneous but cohesively defined group of lysine-targeting methyltransferases that contain a ubiquitously conserved SET catalytic domain (5). A core SET domain consists of two frameworks, one containing a conserved anti-parallel β sheet and the other a variable domain with a knot-like structure, which comprises the active site of the enzyme (33). This SET domain, consisting of around 130 amino acids, was primarily found in *Drosophila melanogaster* genes, with its epigenetic role (34). Whereas its mammalian homologs were the first histone lysine (K) methyltransferases (HKMTs) that can methylate H3 at Lys-9 (35). Now several SET containing HKMTs are recognised that methylate Lys-4, -9, 27 or -36 in histone H3 and Lys-20 in histone H4. Enzymes of this family, namely SETD1A, SETD1B, SETD2, SETD3, SETD5, SETD6, SETD7, and SETD8, mediate the methylation of distinct lysine residues across both histone and non-histone substrates (**Table 2**) (12).

**Table 2:** General features and biological functions of SETD family members.

Member	Substrate (Histone)	Target	Methylation	Functions
SETD1A	H3	H3K4	me1/me2/me3	Activation of transcription, promoter regulation
SETD1B	H3	H3K4	me1/me2/me3	Gene activation, chromatin remodeling
SETD2	H3, Non-histone proteins	H3K36	me3 only	DNA repair, transcriptional progression, RNA splicing, regulation of the immune system
SETD3	Actin non-histone	His73	me1	Cytoskeletal organisation, muscle functioning
SETD5	Histone complexes	Not clearly defined	Uncertain	Regulation of chromatin, neurodevelopment
SETD6	Histone, non-histone proteins	H2AZK7	me1	Transcriptional repression, control of inflammation
SETD7 SET(7/9)	H3, non-histone proteins	H3K4	me1	Cell cycle regulation, transcriptional control
SETD8 (PR-Set7)	H4	H4K20	me1	Cell cycle advancement, DNA replication, chromatin compaction

Details with citations are mentioned in the text.

**Figure 2:**
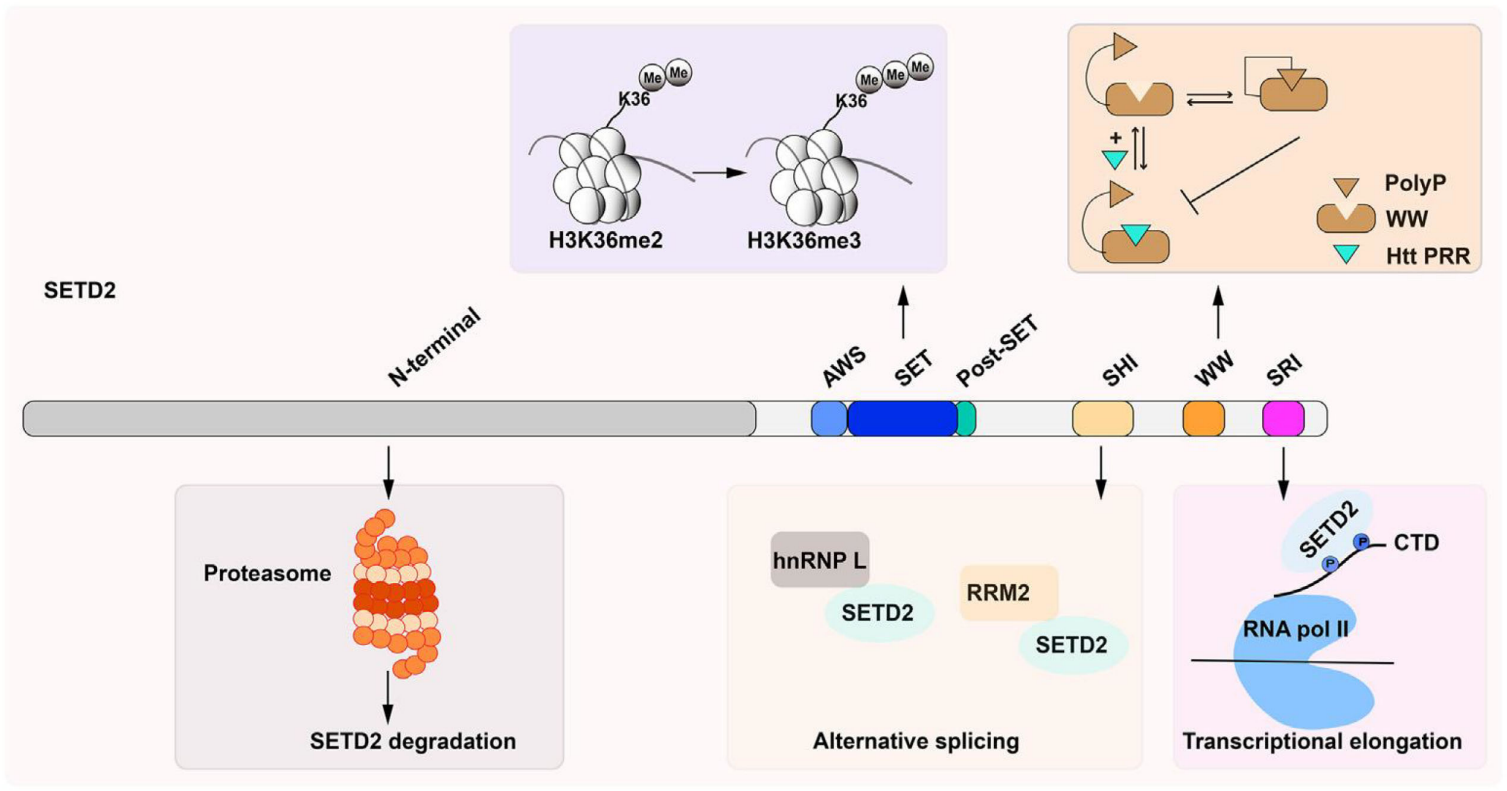
The structure of the SETD2 protein in mammals. The protein has several functional domains: i) the AWS (associated with SET)-SET-PostSET domains, ii) a WW (tryptophan-tryptophan) domain, iii) an SRI (Set2-Rpb1 interacting) domain, iv) an SHI (SETD2-hnRNP interaction) domain, and a large unstructured N-terminal domain (36).

By virtue of selective substrate engagement, each SETD enzyme imprints a distinct posttranslational modification landscape, thereby shaping chromatin structure and modulating transcription (18). SETD1A and SETD1B, for instance, serve as the principal methyltransferases for tri-methylating H3K4, which is involved in the modification of transcribed promoters (32). Among many other markers of immune cells, SETD2 catalyzes the trimethylation of H3K36me3, which is associated with transcriptional inaccuracy and the preservation of the mechanisms for DNA damage and repair (33). This is also related to methylation during differentiation (34), development and physiology of immune cells (35) and methylation of the ACK1 tyrosine kinase (36, 37). The details of the structure of the SETD2 are depicted (**Figure 2**). It is shown that SETD2 has several functional domains. The SET region between AWS and post-SET domains is related to the di- and tri-methylation of H3K36. WW (tryptophan-tryptophan) and SRI (Set2-Rpb1 interacting) domains bind proteins (37). WW interacts with huntingtin-proline-rich region (HTT-PRR) and carries out trimethylation of lysine68 (ActK68me3) in cells, which helps actin polymerization and cell migration (38). SRI mediates SETD2 binding with the RBP1 subunit of RNA polymerase II enzyme, thus helping to methylate actively transcribing genes (39, 40). SETD2 also interacts with heterogeneous ribonucleoprotein L (hnRNP L) and RNA recognition motif 2 (RRM2) and RNA processing machinery via its SHI domain throughout the alternating splicing (41). The N-terminal of SETD2 is involved in its ubiquitin-proteosome pathway (42). Aside from the nuclear environment, both SETD6 and SETD7 have control over the immune system (43, 44). These enzymes methylate certain inactive non-histone molecules to integrate non-histone regulatory protein meshing chromatin and signaling in the NF-κB pathway and to modulate T cell effector functions (45).

## 2 Structural and functional aspects of SETD enzymes

The SETD clade of lysine methyltransferases is distinguished by a SET (Su(var)3–9, Enhancer-of-zeste, and Trithorax) catalytic domain, a conserved element responsible for catalyzing the transfer of methyl groups to the ϵ-amino groups of lysine residues contained within both histone and non-histone protein substrates (5, 34). Notwithstanding its uniform core catalytic architecture, SETD family members manifest extensive heterogeneity in site preference, cofactor binding, and physiopathological engagement (33). This organizational plasticity confers the capability of SETD enzymes to assist as extremely context-specific epigenetic regulators. They effectively integrate pervasive chromatin remodeling signals with immune processes and disease-relevant transcriptional outputs (46).

### 2.1 Conserved SET domain architecture

SETD methyltransferases can be molecularly associated with having a dominantly core SET domain structure, which was first identified as a unit in a complex, root-containing individual units of roughly 30–150 residues and associated with a chromatin modifier complex in *Drosophila* (47). Structural analysis has shown a knot-like scaffold with a central anti-parallel β-sheet with several α-helices configurationally surrounding, constituting a methyl donor binding cleft that sequesters S-adenosylmethionine (SAM) (5). The same cleft simultaneously presents to the ϵ-amino of an incoming substrate lysine, thereby positioning donor and acceptor pairs in the ideal conformation for methyl transfer (48). Pre-SET and post-SET helices with bounding co-translations form domains that frequently stabilize a conserved subject of cysteine-bridged zinc clusters. This, in turn, strengthens global domain stability, increasing the binding site for incoming substrates (5, 47). Modifications in these auxiliary helices account for observed variations in substrate discrimination and provide a molecular basis for the functional and regulatory broadness exhibited by the SETD enzyme cohort (5, 49).

### 2.2 Mechanisms of lysine methylation

**Figure 3:**
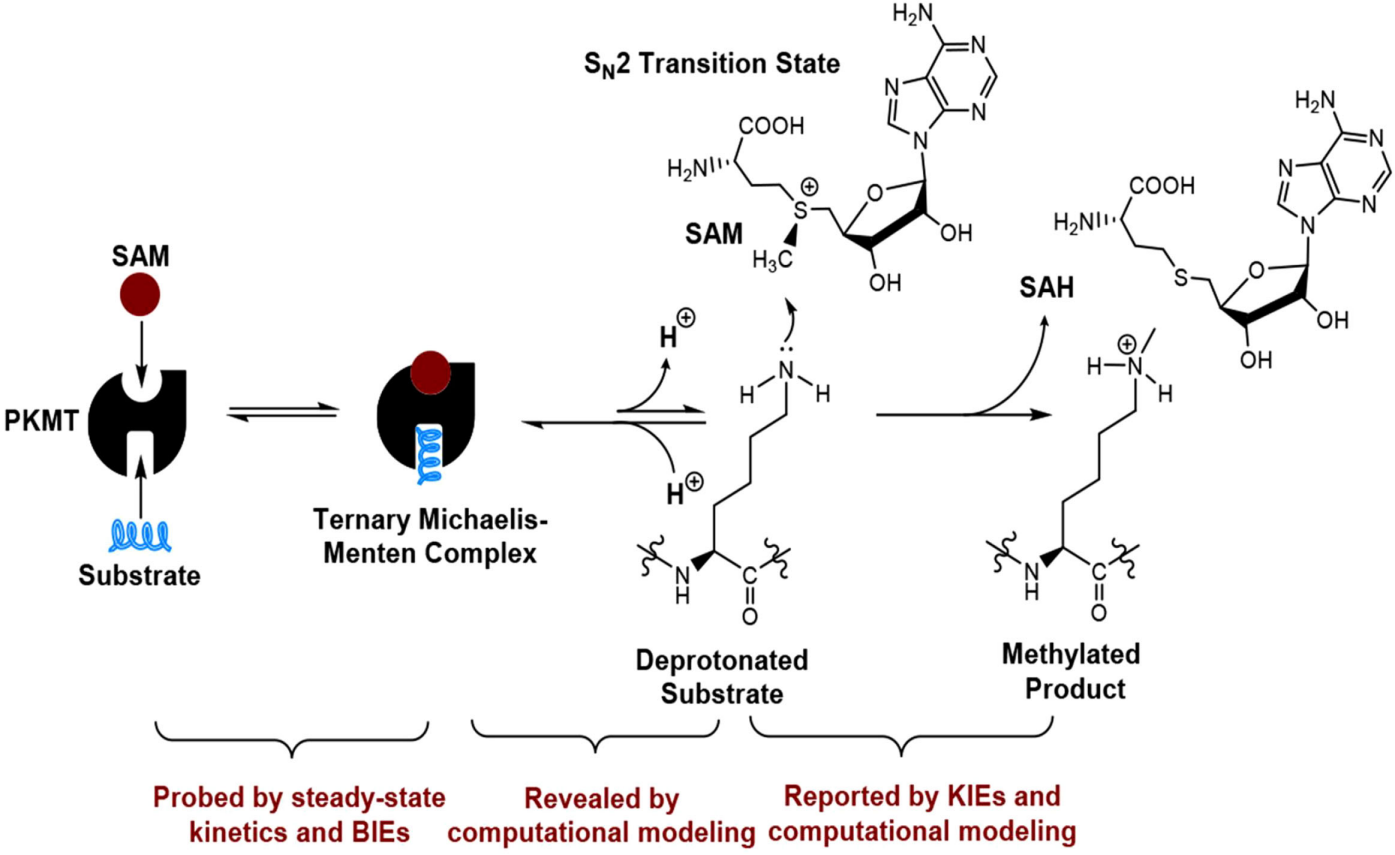
Transfer of a methyl moiety via the SN2 mechanism from the SAM cofactor. Proton abstraction from the ϵ-amino group of lysine yields the SN2 transition state, leading to the formation of methyl-lysine and the concomitant release of S-adenosyl homocysteine (SAH) (50).

SETD methyltransferases mediate lysine methylation through an SN2-type methyl-transfer mechanism that employs SAM as the methyl donor (**Figure 3**). The ϵ-amino group of lysine coordinates to the metal centre (Zn^++^) of the catalytic domain, orienting the nitrogen as a nucleophile and facilitating a concerted displacement of the methyl group (50). Subsequent proton-transfer equilibria result in mono-, di-, or tri-complexes, depending upon the substrate-induced microenvironment and the geometry of the active site tunnel, which is shaped by complementary hydrogen-bond donors and strategically positioned aromatic residues (17). The binding channel is optimised not only for lysine side exposure but also for differential ion binding, strictly suppressing di-methylation unfolding and packing as a rigid isobutanol centre (50, 51). Clinically, each lysine methylation level provides a stratified cellular message: for instance, the mono-methylation of H3K4 is a prevalent enhancer signature, while tri-methylation of the same position marks transcriptionally active core promoters (6, 17). Conversely, tri-methylation of H3K9 and H3K27 is dominantly inscribed on heterochromatic domains and gene-silencing loci (35). Consequently, family-wide SETD catalysts have evolved to subscribing not solely to a catalytic agenda, but to imprinting a conserved, molecular 3D alphabet of histone and protein methylation. It thus guarantees immune transcriptional landscapes with enduring epigenetic memory (50).

### 2.3 Histone and non-histone substrates

Although initially defined as histone methyltransferases, the SET domain family has been shown to catalyze methylation of numerous non-histone targets, thus broadening the scope of their regulatory activity beyond the nucleosome (46, 52). SETD6, for example, monomethylates the RelA subunit of NF-κB, resulting in downstream attenuation of pro-inflammatory transcription (44). In contrast, SETD7 methylates p53 and STAT3, thereby modulating apoptosis and cytokine signaling, respectively (53, 54). SETD8 is the only enzyme identified to mono-methylate p53 on Lys382 (p53K382me1), resulting in the inhibition of its pro-apoptotic and growth-arresting roles (55). It has been suggested as the initial step in the start of inflammation-induced colorectal cancer and, therefore, is a diagnostic marker and therapeutic target (56). These non-histone modifications expand the functional repertoire of SET domain enzymes, enabling the targeted modulation of immune signaling, stress-response circuitry and chromatin-linked transcriptional control (19). By simultaneously modifying histone and non-histone substrates, the SETD family operates at the nexus of epigenetic and cellular signaling frameworks, thereby establishing a coordinated interface between transcription and inflammation, notably in autoimmune pathology (57, 58).

## 3 SETD proteins in chromatin rebuilding and gene regulation

### 3.1 Histone modification and transcriptional achievements

Histone lysine methylation, catalyzed by SETD enzymes, is an integral component dictated by PTMs. In this regard, SETD1A and SETD1B-augmented trimethylation of histone H3 lysine 4 (H3K4me3) bind gene promoters and regulate transcriptional co-activators and RNA polymerase II activities (59). SETD2-catalyzed methylation of lysine 36 on histone H3 (H3K36me3), and it sets transcriptional elongation along with co-transcriptional processes linked with DNA repair (46, 60). The SETD7 and SETD8 methylate H3K4me1 and H4K20me1 and affect transcription initiation and termination (54). The SETD enzymes also methylate non-histone substrates and regulate the expression of specific genes involved in the innate and adaptive immune responses (61). Likewise, SETD8 has been modulated in the regulation of angiogenesis through HES-1 in endothelial cells (62).

### 3.2 Crossover with other epigenetic transformers

Both synergistic and antagonistic links with a variety of other epigenetic regulators have been identified by SETD enzymes. One example is H3K4 trimethylation, catalyzed by SETD1 complexes, that coexists with HATs to generate open chromatin conformations that enable the assembly of active transcription complexes (59, 63). Another mechanism is detected when SETD2 catalyzes H3K36 trimethylation, which inhibits the recruitment and buildup of polycomb repression complexes. It thereby safeguards active loci from H3K27 repressive actions (46, 60). SETD7 carries out the methylation of both histone and non-histone substrates, including the methylation of DNA methyltransferases and consequently affecting the regional distribution of DNA cytosine modification (54). These studies reflect that SETD-derived changes have a wider epigenetic impact. The transcriptional changes in immune cells are found to be both stiff and plastic in their response to environmental stimuli.

### 3.3 Convergence with signaling pathways

Additionally, SETD methyltransferases dynamically interface with pathways governing immune activation and inflammatory responses (63, 64). For example, SETD6 catalyzes RelA methylation. It also modulates the transcriptional output of the NF-κB cascade of events, which is a principal regulator of inflammatory changes (45). SETD7 methylates STAT3 and the Th17 family associated with interleukin signaling (54). H3K36 trimethylation by SETD2 profoundly influences immune functioning by affecting transcriptional memory, responsive functions and damage to immune genomic integrity (60). The methylation processes are biochemically and epigenetically encoded transcriptional responses that include extracellular signals like cytokines, pathogen-associated molecular patterns (PAMPs), and other stress response factors (46). Nonetheless, SETD members exhibit immune plasticity with activating signals involved in epigenetically making responsive changes. It therefore has therapeutic potential in the chronic inflammatory immune diseases (44).

## 4 The immunological profiles of SETD family members

### 4.1 SETD1A/B: H3K4 methylation and T-cell differentiation regulations

**Figure 4:**
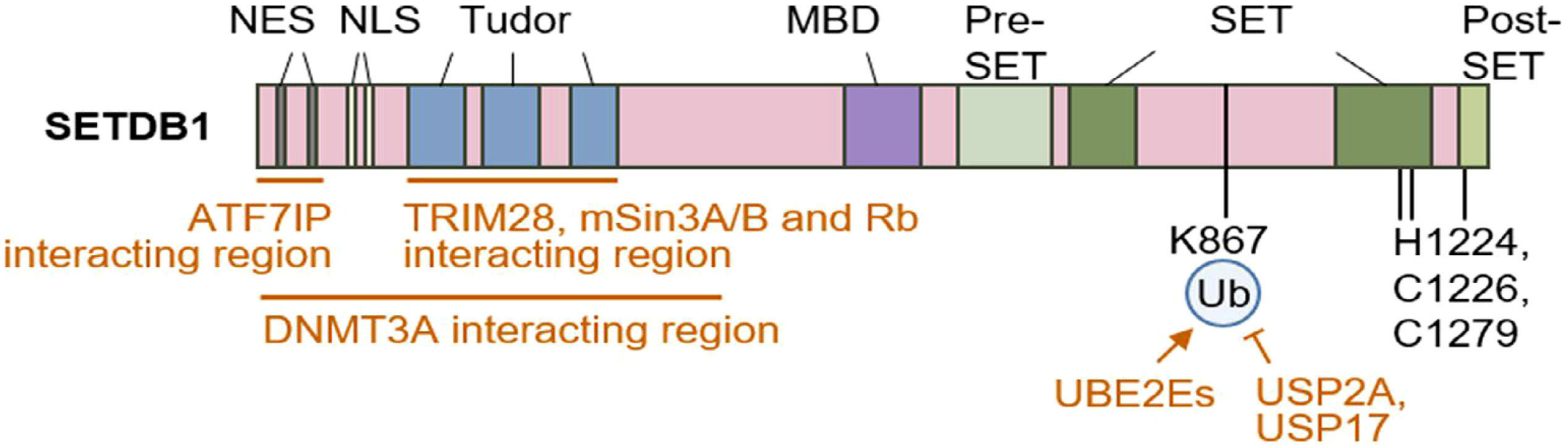
Schematic representation of human SETDB1 and its interaction networks. Distinct domains are accented by a color code. MBD denotes the methyl-CpG binding domain; NES and NLS mark the nuclear-cytoplasmic export and nuclear localization sequences; the SET domain indicates simultaneous scaffolding and catalytic activities. Monoubiquitination of lysine 867 occurs through the UBE2E E2 family, followed by deubiquitination led by USP2A and USP17. Efficacy of the catalytic SETDB1 function is abrogated by amino acid substitutions at histidine 1224, cysteine 1226, or cysteine 1279 (72).

The SETD1A and SETD1B are vital members of the COMPASS complex, causing trimethylation of H3 at lysine 4, which is indicative of transcriptionally active chromatin (65–68). In T lymphocytes, the H3K4me3 is orchestrated primarily by SETD1A and SETD1B. It is superimposed at lineage-determining loci and subsequently shapes the occupancy and activity of key transcription factors, including T-bet in the Th1 subset, GATA3 in Th2 cells, and RORγt within Th17 cells (**Figure 4**) (69). Deviation from tightly controlled SETD1-mediated trimethylation of H3K4 engenders soft control of lineage fidelity, undermining T-cell homeostasis and triggering the emergence of autoimmune manifestations. This thereby indicates that only narrowly defined boundaries of H3K4 methylation permit the maintenance of balanced adaptive immunity (59, 70). A regulation of the NF-κB pathway involving SETD1 in periodontal inflammation via H3K4 trimethylation has also been demonstrated (71).

### 4.2 SETD2: H3K36 trimethylation in cytokine signaling and DNA repair

The sole methyltransferase responsible for the trimethylation of histone H3 at lysine 36, SETD2 (KMT3A and SET2), protects the 3-methyl mark in actively elongating transcription units (60). Within hematopoietic cells, SETD2 acts as the molecular fulcrum that reconciles pro-inflammatory signaling within the interferon and interleukin signaling cascades and the transcript elongation machinery (73). Concurrently, through the H3K36me3 mark, SETD2 orchestrates the recruitment of DNA repair complexes, most notably the homologous recombination machinery, to the locus of double-strand breaks. It thereby embeds chromatin-imposed genome surveillance within the activation program (46). In-frame mutations occlude SETD2 enzymatic activity that disrupts efficient T-cell receptor transduction. It renders repair of double-strand breaks in DNA and promotes inflammatory environments as well as malignant expansion. It establishes a unified nexus of transcriptional fidelity and genomic maintenance in adaptive immunity (46, 74).

**Figure 5:**
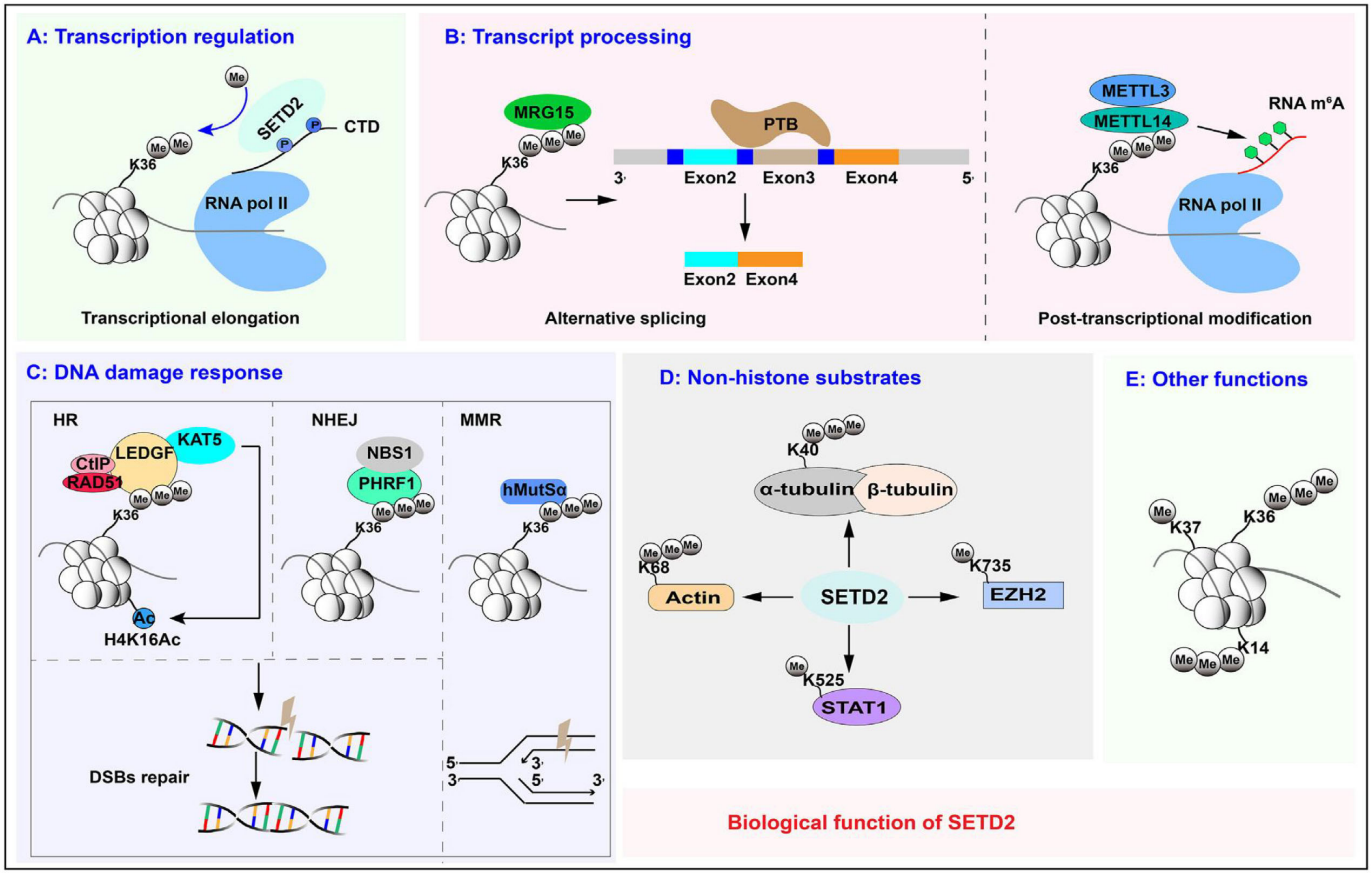
Cumulative illustration of the physiological and pathological roles of SETD2, depicting orchestrated control of the DNA damage response, roles in splicing regulation, and deposition of methylation on histone and RNA species, couplings to transcription-coupled histone modifications and diverse post-translational changes (36). **(A)** Transcription regulation. **(B)** Transcript processing. **(C)** DNA damage response. **(D)** Non-histone substrates. **(E)** Other functions.

A recent study has shown that SETD2 drives injury in failing hearts, and targeting this enzyme may prevent heart injury (75). The biological functions of SETD2 are schematically summarized below (**Figure 5**) (36). In a recent publication, SETD2 has been reported to be involved in the regulation of inflammation-induced trained immunity of macrophages. Two mechanisms have been suggested regarding the SETD2 link to the regulation of pro-inflammatory and metabolic pathway reprogramming (76). SETD2 augments glycolytic and inflammatory pathway component genes via enhancer-promoter looping without any enzymatic role. The second mechanism involves increased promoter-associated H3K9 methylation and repression of interferon response pathway genes (77, 78). SETD2 has been verified as an epigenetic driver in the molecular mechanisms of recurrence and metastasis of renal cancer (79) and prostate cancer (80). SETD2 epidermal deficiency has been involved in the promotion of cutaneous wound healing through the activation of AKT/mTOR signaling pathway and has been suggested to be actively involved in psoriasis (74, 81).

**Figure 6:**
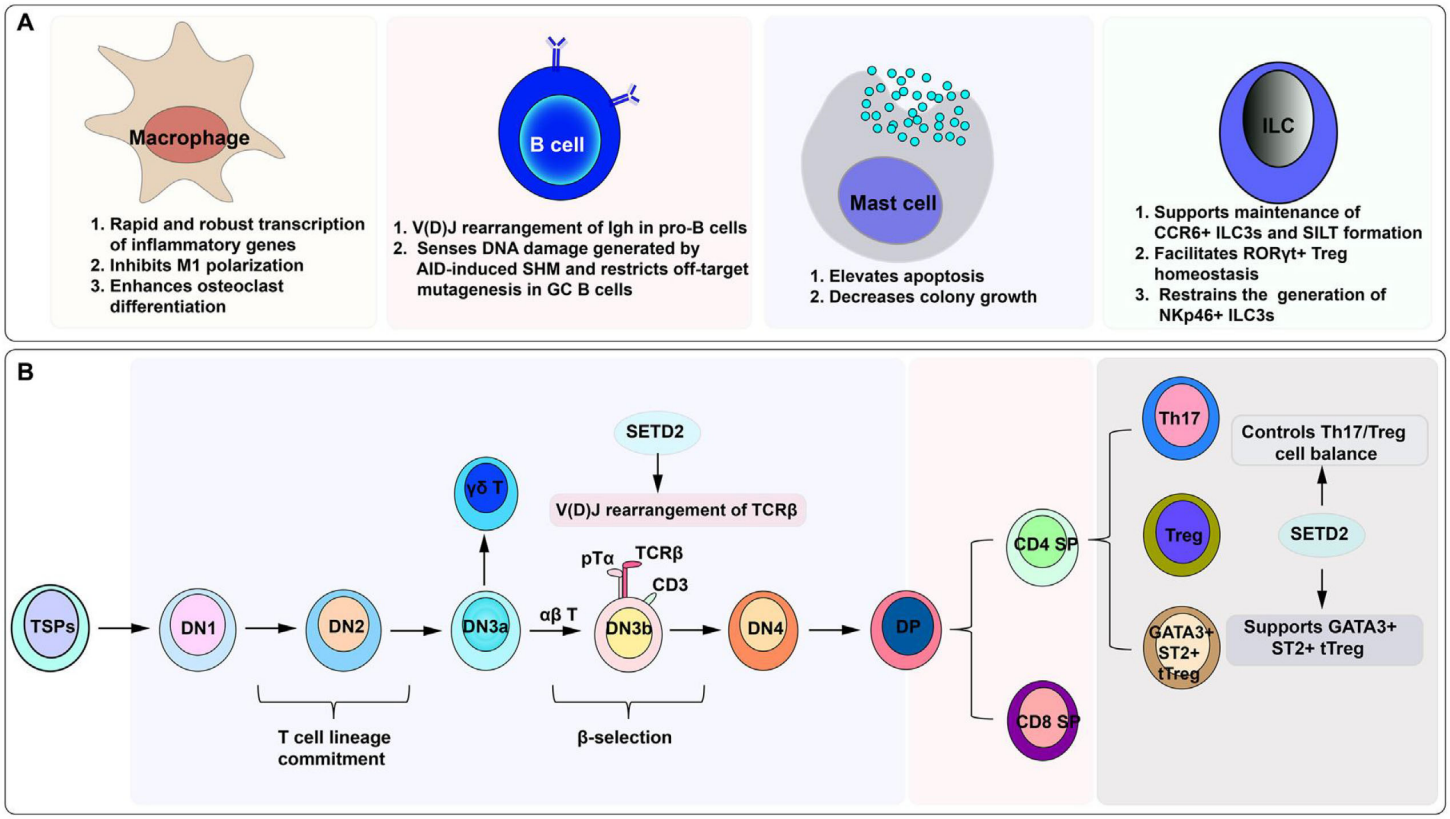
An integrated overview of the impact of SETD2 on distinct immune cell lineages. SETD2 is required for the specification of immune cell lineages and for the subsequent refinement of their biological activities. SETD2, SET domain-containing protein 2; AID, activation-induced cytidine deaminase; GC, germinal centre; SHM, somatic hypermutation; TSP, thymus-seeding progenitor; DN, double-negative; DP, double-positive; SP, single-positive (36).

SETD2 operates as a bona fide tumor suppressor by enforcing p53-dependent transcriptional fidelity, coordinating transcriptional elongation, and regulating splice-site choice in RNA splicing (82–84). The cytokine and chemokine response of this tumor suppressor is reflected by the recent identification of SETD2 mutations in the genomes of human breast cancer specimens (82, 83). SETD2 inactivating alterations are also demarcated in clear-cell renal cell carcinoma (85, 86) and non-small-cell lung carcinoma (**Figure 6**) (87).

### 4.3 SETD3: actin methylation and cytoskeletal dynamics

**Figure 7:**
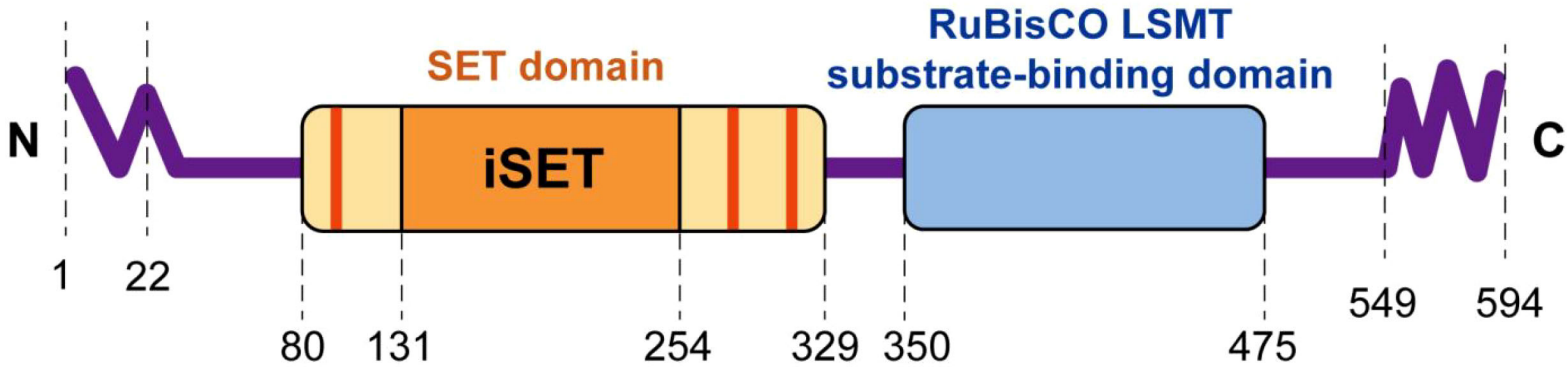
Structural details and characteristic domains of human SETD3 (93). The human SETD3 protein contains 594 amino acid residues with a SET domain of 80–329 residues. The inserted region (iSET) consists of 131–254 residues. Red bars in the SET domain indicate S-adenosyl methionine binding sites. The green-highlighted domain resembles chloroplast-localised RuBisCO (Rubisco) large subunit methyltransferase (LSMT) binding domain. It is suggested that this domain may act as the substrate recognition and binding domain of SETD3. The wavy -N and -C ends indicate disordered structures.

Distinct from other SETD family methyltransferases, SETD3 selectively methylates non-histone targets, predominantly by modifying β-actin at histidine residues (88). This methylation alters the stability of the filamentous actin cytoskeleton and reorganizes the supramolecular architecture of the immune synapse, thus enabling the precise presentation of antigen (89). SETD3 has been reported to regulate alternative splicing of the mRNA through interacting with heterogeneous nuclear ribonucleoproteins (hnRNPs) (90, 91). Loss-of-function models of SETD3 demonstrate weakened T-cell-antigen-presenting cell coupling, diminished leukocyte translational motility, and impaired clearance of intracellular pathogens, thereby situating this enzyme at the nexus of actin sorting and adaptive immunity. The structural details of the protein are given in **Figure 7**, while the reported activities and proposed functions are summarized in **Table 3** (92, 93).

**Table 3:** A summary of SETD3 activity across different signaling pathways (93).

Process	SETD3 activity	Proposed functions	References
Actin polymerization	Methylation of H73 in β-actin	Stabilization of actin monomers; promotion of actin polymerization	(94, 95)
Enterovirus pathogenesis	Unknown	Support of viral genome replication; interaction with viral protease 2A	(94)
Epigenetic regulation of chromatin	Methylation of core histones, including H3	Regulation of gene expression	(96–98)
Response to hypoxic conditions	Methylation of FoxM1	Increase in VEGF expression; promotion of angiogenesis	(99)
Carcinogenesis	Unknown	Regulation of cancer development and progression	(97, 99–107)

### 4.4 SETD4: proliferation of macrophages and onco-immunological biomarkers

An abnormal expression of SETD4 has been noticed in various types of cancers, including colorectal cancer, where its upregulation determines its prognostic risk factor (108). Poor methylation of the SETD4 promoter in colorectal cancer patients predicts shorter survival times with effects on G2M checkpoint, mitotic spindle pathways. However, negative effects of SETD4 were observed in immune cell infiltration, including B cells, CD8 T cells and macrophages (108). A recent study in *SETD4* knockout mice has demonstrated that SETD4 is involved in mediating EGFR signalling in the proliferation of bone marrow-derived macrophages (BMMs), where it carries out the methylation of lysine at multiple sites in these cells (109). The same research group reported earlier the role and applications of SETD4 in the proliferation, migration, angiogenesis, myogenic differentiation and genomic methylation of bone marrow mesenchymal stem cells (110).

SETD4 is implicated in lysine methyltransferase activity in the cytosol and nucleus in breast cancer and has been suggested as a diagnostic and therapeutic target (111). SETD4 has been implicated in the positive regulation of IL-6 and TNF-α expression in Toll-like receptor (TLR)-agonist-stimulated macrophages by directly activating H3K4 methylation (76, 108). The other functions of SETD4 include its association in breast cancer stem cell quiescence (112), conferring cancer stem cell chemoresistance in non-small cell lung cancer (113), inhibiting prostate cancer by transcription repression and cell cycle arrest (114), role in pancreatic development from induced pancreatitis (115), cardiac neovascularization of capillaries organ development and regeneration (116), promoting recovery of hematopoietic failure in mouse (117) and regulating cell quiescence and trimethylation of H4K20 (118). The role of SETD4 in inflammatory responses has also been demonstrated in the methylation of TBK1 in interferon signaling (119).

### 4.5 SETD5: transcriptional repression and neuro-immune regulation

**Figure 8:**
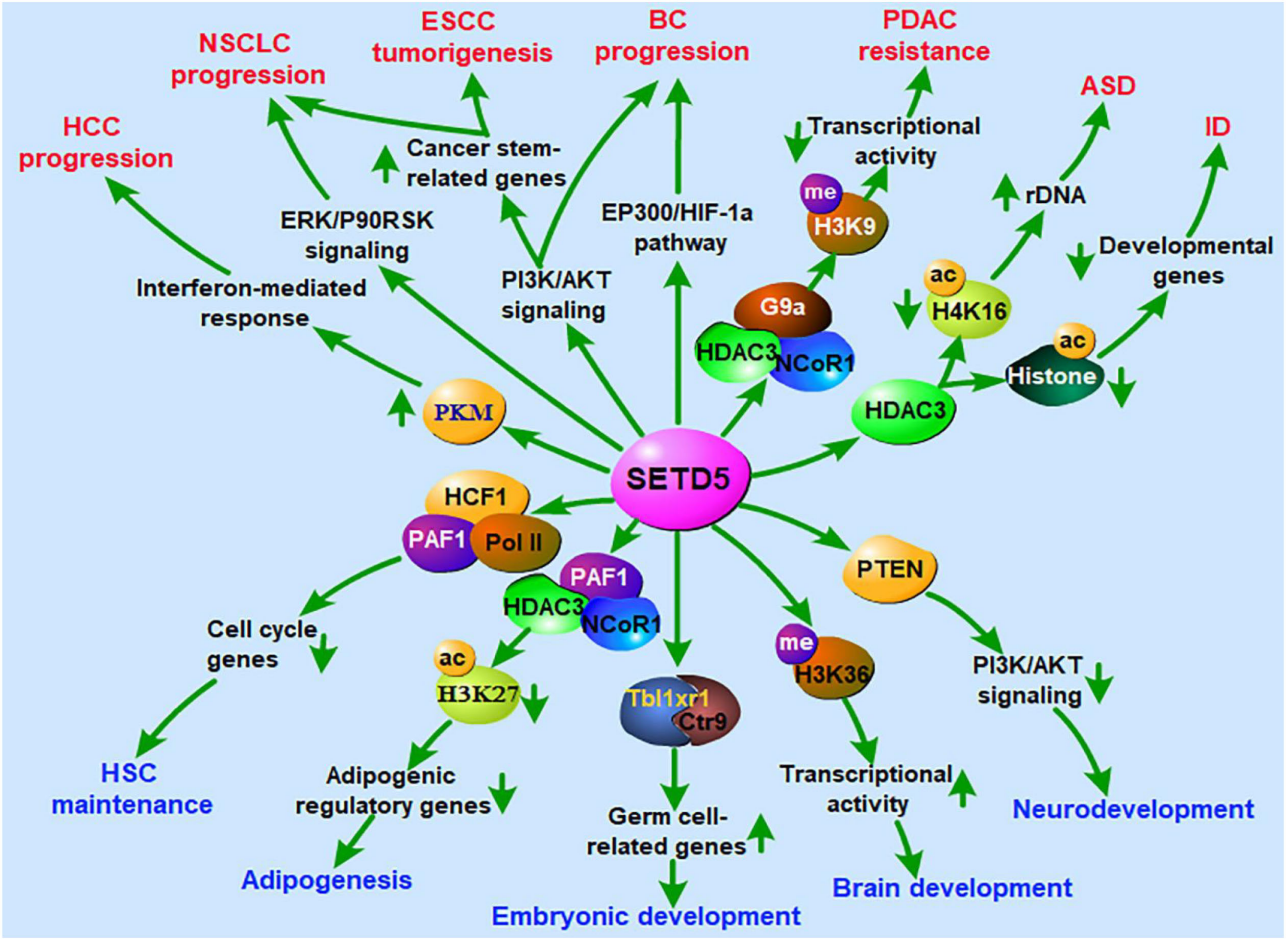
A summary of events of SETD5 regulating the development of the nervous system, embryonic development and carcinogenesis. ASD, autism spectrum disorder; BC, breast cancer; ESCC, esophageal squamous cell carcinoma; HCC, hepatocellular carcinoma; HSC, hematopoietic stem cell; ID, intellectual disability; NSCLC, non-small cell lung cancer; PDAC, pancreatic ductal adenocarcinoma (120).

Although SETD5 was initially characterized as a transcriptional repressor defective in neurodevelopmental disease, its list of cellular audiences now also includes the immune system (120). The enzyme purportedly catalyzes trimethylation of H3K4, but persistent debate questions the substrate specificity and mechanism (121). Regardless, SETD5 multi-molecular complexes modify the higher-order configuration of histones and non-histones, often in the context of microglial interactions with neurons (120). When SETD5 dosage falls abnormally low or high, microglia and peripheral leukocytes overcommit inflammatory programs that fuel neurodegenerative disease. It thus observes fundamental neuro-immune crosstalk anchored in an epigenetic scaffold (32). Studies have demonstrated a considerable involvement of SETD5 in various classes of cancers, like lung cancer (122, 123), leukemia (29), breast cancer (124), pancreatic cancer (125), and autism (126–129) and epilepsy (130, 131) with related brain development and neuronal disorders (132) as summarized (**Figure 8**).

### 4.6 SETD6: NF-κB homeostasis in autoimmunity and inflammation

SETD6 restricts NF-κB activity through site-specific monomethylation of the hyperphosphorylated RelA tail (45). The resulting attenuation of NF-κB transcriptional activity constitutes a feedback checkpoint that limits programmed inflammatory responses (32). In macrophages and dendritic antigen-presenting cells, SETD6 calibrates the duration and intensity of TLR and other pattern-recognition receptor signaling by repressing excessive IL-6 and other pro-inflammatory molecules (32, 76). Pharmacologically compromised SETD6 expression unleashes terminal NF-κB super-activation and autonomous JAK-STAT with sufficient vigor to generate clinical and laboratory features resembling human systemic autoimmunity (45).

**Figure 9:**
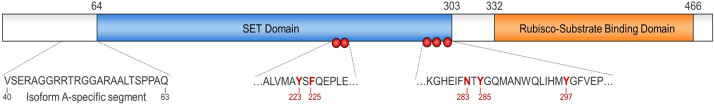
Domain architecture and assembly of the SETD6 protein. SETD6 comprises two key functional elements: the SET domain (blue) and a substrate-binding module resembling the Rubisco ligand-binding domain (orange). The residues constituting the active site are indicated by red spheres. Isoform A contains a 23-residue peptide extension that is absent in isoform B (133).

SETD6, therefore, exhibits features of a dynamic epigenetic rheostat within the innate immune system and constitutes an actionable target in antiautoimmunity strategies that capitalize on excessive NF-κB signaling (45). RAD18 is a RING-finger E3 ubiquitin ligase enzyme linked with the DNA damage response (DDR) pathway in the post-replication repair during the G1 stage. A positive correlation between SETD6-mediated methylation of RAD18 has been demonstrated, which plays a vital role in mutagenesis and the cellular response to DNA damage (44). A comprehensive review of the SETD6 methyltransferase has been published with details of the structural and functional organisation of this molecule (**Figure 9**) (133).

### 4.7 SETD7: monomethylation at the tumor-immune interface

SETD7 (KMT7, SET7, SET9, SET7/9) preferentially targets and monomethylates the N-terminal H3K4 peptide in a reconstituted system (134, 135). In addition to canonical histones, SETD7 selects a diverse array of nonhistone substrates and transcriptional cofactors, including p53, estrogen receptor alpha (ERα), retinoblastoma protein (pRb), STAT3, hypoxia-inducible factor alpha (HIF-α), forkhead box factor O3 (FOXO3), and DNA methyltransferase 1 (DNMT1). Consequently, SETD7 orchestrates multiple layers of transcriptional control and lineage-committed cell differentiation (54, 136–143). Extensive analyses furthermore established that SETD7 directs monomethylation of lysine 310 in the NF-κB p65 subunit, thereby modulating the transcriptional strength and localisation of this signaling pathway (144, 145). Several studies have encouraged targeting SETD7 as a novel pharmacological modality in the context of type 2 diabetes mellitus (146) as well as cardiovascular diseases like atherosclerosis (147, 148).

**Figure 10:**
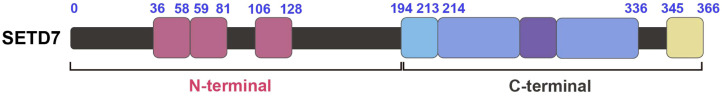
Structural map of SETD7, displaying the assembly of N- and C-terminal domains. The N-terminal domain comprises a triple antiparallel β-sheet motif; adjacent motifs are defined as MORN 1, 2 and 3 (colored in medium orchid). The C-terminal domain is organized as three β-sheets; in succession, the SET domains are displayed in medium slate blue, while the distribution of the subsequent and preceding SET domains is colored in khaki and light sky blue, respectively. An additional motif in the C-terminal domain is designated as i-SET (medium purple). Abbreviations: MORN, membrane occupation and recognition nexus (149).

Embedding these parallel mono- and poly-methylation modules within a unified SETD7 signaling framework allows the kinase to modify signal-integrated transcription programs in a stem-immune and tumor-centric circuit (149). In T helper cells, cytosolic STAT3 pK4-specific monomethylation enforces RUNX- and RORα- reliant Th17 commitments and restricts IL-17 and IL-21 homing (53). In adjacent malignant tissue, SETD7-modified CTLA-4, PD-1, and other checkpoint modules amplify inhibitory and diminish activating programs, dampening cytotoxic therapy responsiveness (54). SETD7, therefore, constitutes a dual and centralized rheostat receptor regulating both antitumor immunity and acquired immune resistance in infiltrated inflammation (150). SETD7 is downregulated in cardiomyocytes under hypoxia and reoxygenated stressed states, antagonizing the upregulated KDM1A. It thereby involves the regulation of methylation patterns at the K151 site of the ATG15L1 (151). Many biological processes and signaling pathways have been regulated by the SETD7 enzyme in correlation with cancer (152). SETD7 is a 41 kDa protein present in both nuclear and cytosolic compartments with diverse functions of methylation in histone and non-histone proteins (**Figure 10**) (149).

### 4.8 SETD8: H4K20 monomethylation, immune checkpoint and DNA repair

SETD8 represents the singular enzyme responsible for the monomethylation of histone H4 at lysine 20, a PTM intimately associated with chromatin condensation and the preservation of genomic integrity (19, 153). SETD8 involvement has been documented in psoriasis (81) and vascular calcification and atherosclerosis (154, 155). In a broader context, the substrate specificity of SETD8 extends beyond histones to include the tumor suppressor protein p53 and other determinants of the DNA damage response and cell-cycle checkpoint pathways (156, 157). Within the hematopoietic compartment, SETD8 modulates the clonal expansion and survival of T and B lymphocytes after antigenic challenge. Of particular translational relevance, aberrant SETD8 activity has been shown to condition the expression of inhibitory immune checkpoints, notably programmed cell death ligand 1 (PD-L1). It thereby connects chromatin remodelling to the acquisition of immune-resistance phenotypes in neoplastic disease (64). Further, the regulation of angiogenesis in human umbilical vein endothelial cells has been demonstrated by SETD8 (62). Collectively, these observations position selective inhibition of SETD8 as a rational therapeutic approach to potentiate antitumor immune responses and re-establish immune homeostasis in inflammatory contexts (57). Nonetheless, SETD8 has exhibited elevated expression across multiple malignancies, encompassing non-small and small cell lung carcinomas, hepatocellular carcinoma, pancreatic adenocarcinoma, bladder carcinoma, and chronic myelogenous leukaemia (12, 158).

## 5 SETD enzymes in inflammatory and autoimmune disorders

### 5.1 Rheumatoid arthritis and chronic inflammation

**Figure 11:**
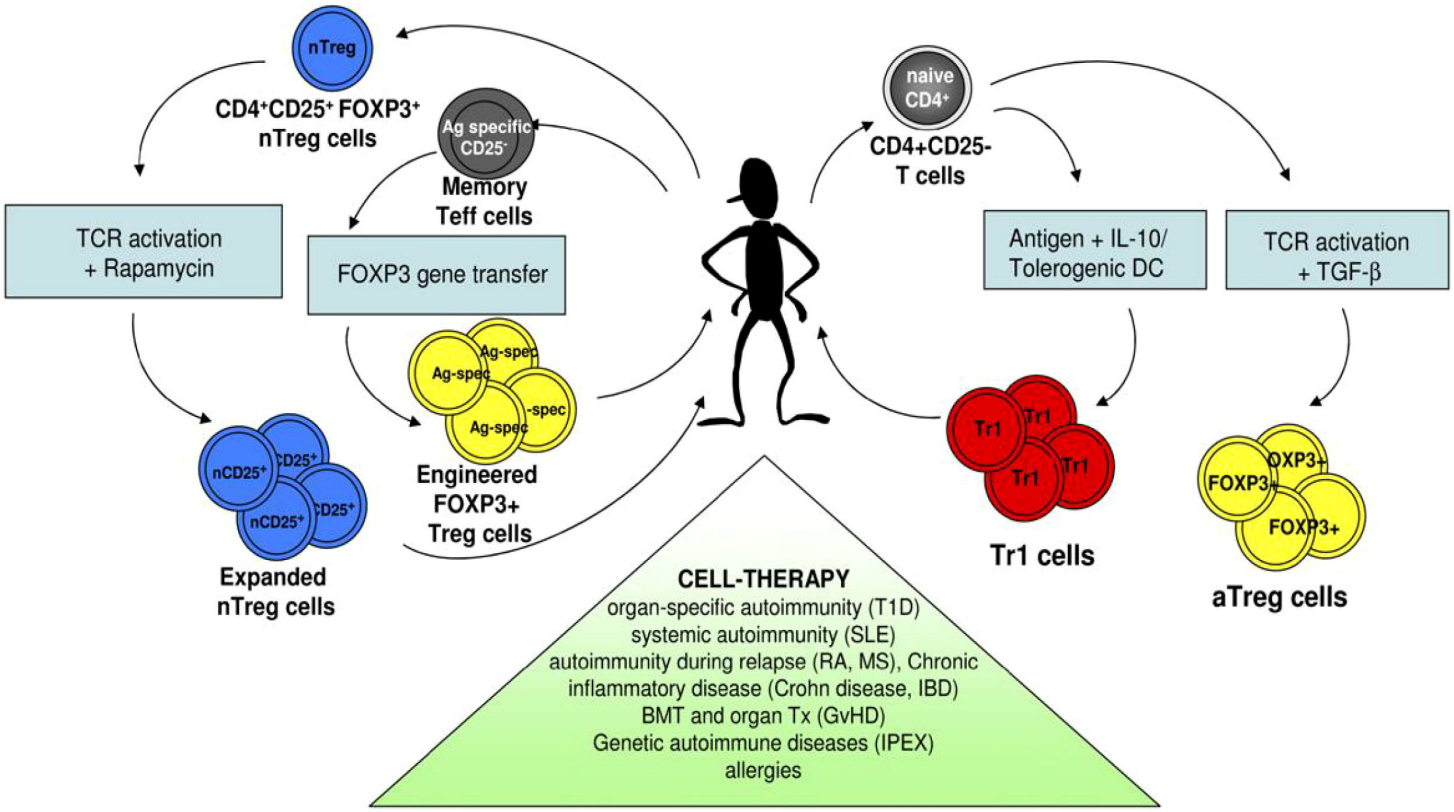
Methodologies for generating ex vivo regulatory T cells employed in the therapeutic management of SLE, RA, MS, IBD, organ transplant tolerance and allergic diseases (165).

**Figure 12:**
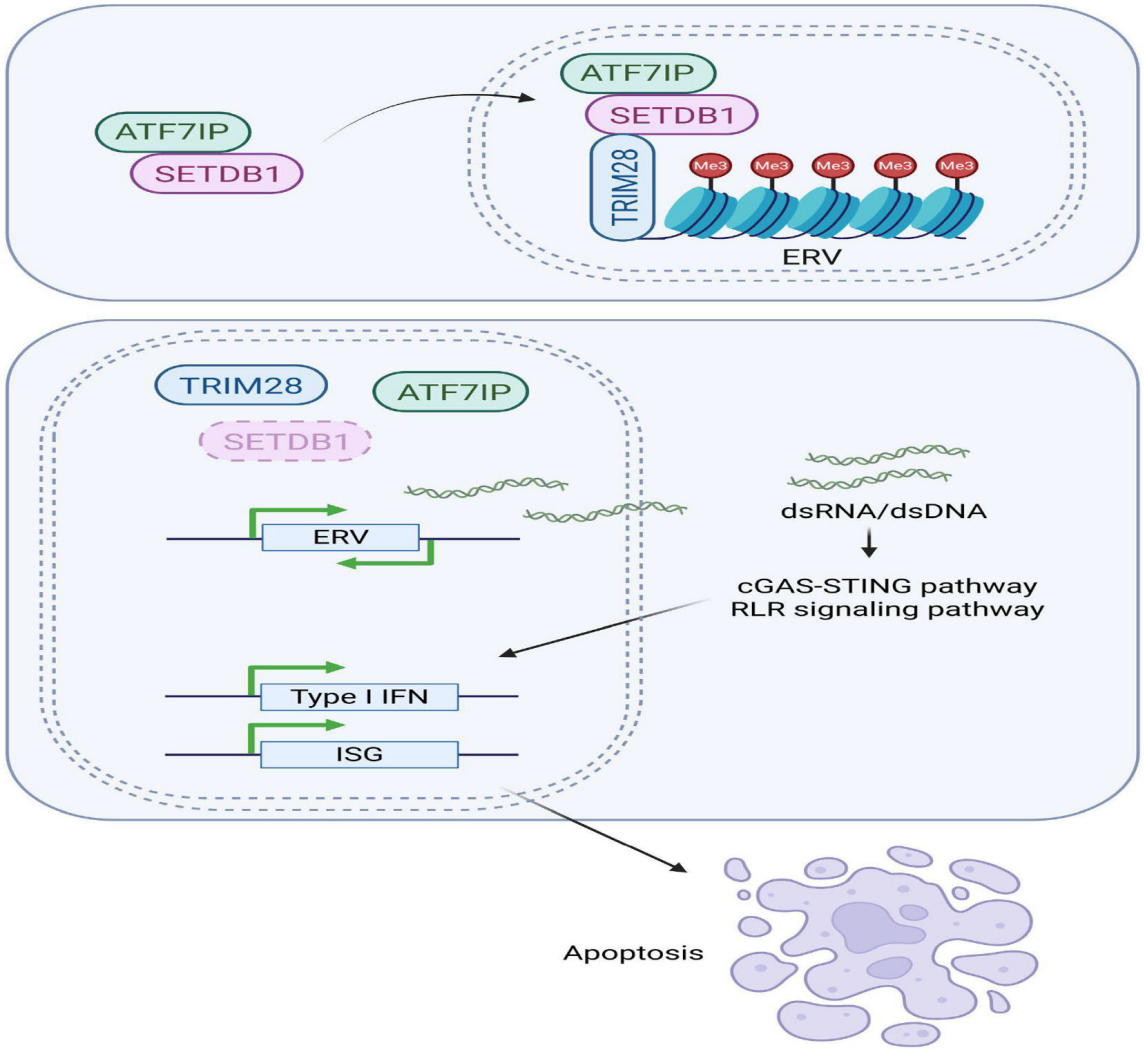
Illustration of the SETDB1-mediated regulation of gene expression. SETDB1, a methyltransferase, is recruited to chromatin through binding to ATF7IP, where it catalyzes H3K9 trimethylation in partnership with the chromatin adaptor TRIM28, constituting a repressing complex that secures transcriptional silencing. When SETDB1 is knocked out, transcriptional depression of endogenous retroviral sequences (ERVs) ensues, giving rise to double-stranded nucleic-acid species, dsRNA or dsDNA. Detection of these molecules by the cGAS-STING axis or the RIG-I-like receptor (RLR) pathways induces a type I interferon signature, which in turn activates a cohort of interferon-stimulated genes (ISGs). The amplification of this inflammatory milieu ultimately triggers programmed cell death pathways (72).

**Figure 13:**
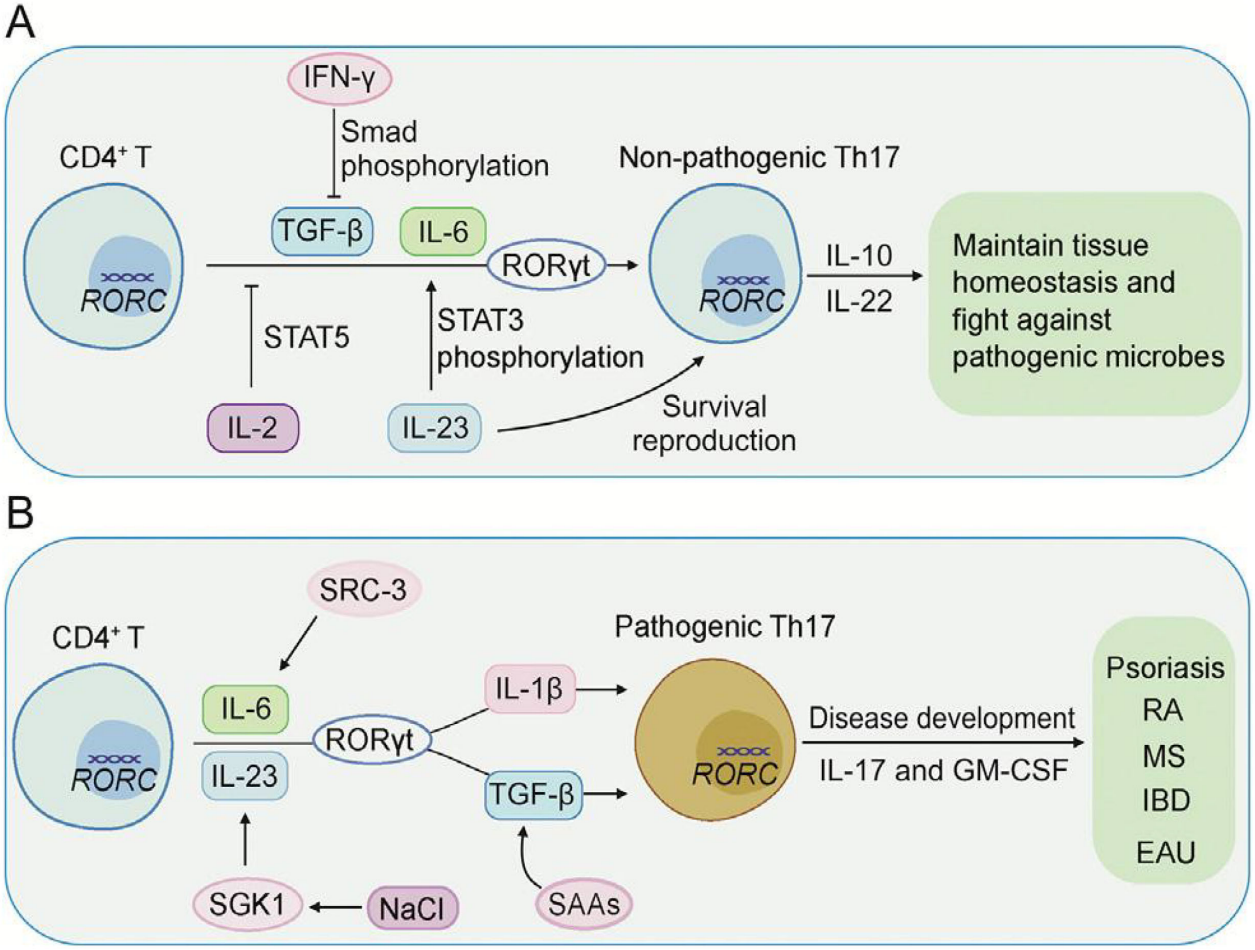
CD4þ T-cell differentiation mechanisms leading towards distinct Th17 subsets. **(A)** The combined action of IL-6 and TGF-β facilitates T-cell precursors towards Th17 differentiation. Within this framework, IFN-γ, IL-23, IL-2, IL-4, and an array of secondary cytokines exert modulatory influence to sustain a Th17 phenotype devoid of pathogenic potential. **(B)** In an alternate signaling milieu, combinations of IL-6, IL-23 and either IL-1β or TGF-β facilitate the generation of pathogenic Th17 cells. This subset emerges concomitant with elevated transcription of IL-17 and GM-CSF, cytokines operative in orchestrating the pathophysiology underlying psoriasis, RA, MS, IBD and experimental autoimmune uveitis. The transcription factor RORγt, in concert with SRC3 and SGK1, anchors this pathogenic program, while coincident expression of serum amyloid A proteins serves as an inflammatory hallmark (69).

Rheumatoid arthritis (RA) is characterized by persistent synovial inflammation and autoantibody production, both of which are shaped by epigenetic misregulation, including the regulatory mechanisms of nucleic acid methylation and miRNA expression (159). Dysregulated SETD1A/B activity and the resultant alterations in histone H3K4 methylation have been linked to the aberrant expression of pro-inflammatory cytokines, including IL-6 and TNF-α. Under homeostatic conditions, SETD6 represses excessive NF-κB activity through the methylation of RelA, yet in RA, diminished SETD6 function releases this inhibitory constraint, which in turn enhances NF-κB-mediated inflammatory signaling (32, 160). Moreover, aberrant expression of SETD7 has been associated with excessive differentiation of Th17 cells, which reinforces IL-17–driven synovial inflammation (53). Collectively, these observations provide compelling evidence that SETD enzymes are key effectors of chronic immune activation and joint destruction in RA, osteoarthritis and cartilage damage (61). In this context, **Figure 11** elaborates the common convergent immunological nodes, predominantly the Th17 pathogenic differences in inflammatory and autoimmune diseases mentioned herein. Whereas, **Figure 12** describes regulatory T-cell-based tolerance monitoring strategies that persist across several autoimmune and inflammatory illnesses like RA, SLE, MS and IBD. Both these figures encompass their part in conceptual synthesis and are not disease-specific (Sections 5.1-5.4) (**Figures 13**, 12).

### 5.2 Systemic lupus erythematosus

SLE is a chronic autoimmune disease affected by environmental factors and epigenetic modifications through DNA, RNA and protein modifications (161). It is characterized as a multisystem autoimmune disorder orchestrated by dysregulated B-cell activation and subsequent synthesis of pathogenic autoantibodies (162). Comprehensive epigenomic profiling has documented selective derangements in trimethylation of H3 lysine residues 4 and 36. It thereby implicates the SET1 and SET2 methyltransferase complexes in the pathogenic circuitry. More pronounced defects in SET2, through its normative engagement in DNA-damage repair, catalyze the accrual of unrepaired DNA lesions, which catalyze type I interferon (IFN-I) production, a hallmark pathogenic pathway in lupus (163).

Alongside others, SETD7-enhanced dynamics of the regulatory layer of the STAT3 transcription factor drive even further the proliferation of autoreactive B and T lymphocytes (54). As a whole, these data build toward a working hypothesis in which the intentional alterations of SETD methyltransferases interweave chromatin restructuring, repair, and lymphocyte overactivation in the developmental environment of SLE (164). More work on the understanding of the mechanisms of epigenetic alterations in the occurrence and development of SLE is suggested for the effective treatment of the disease (161).

### 5.3 Multiple sclerosis and neuroinflammation

Multiple sclerosis (MS) is a chronic, demyelinating neurodegenerative inflammatory ailment affecting 2.9 million people globally, affecting young and adults, dominating in females than males (166). The initiation of MS is concomitant with a greater probability of developing into primary and then secondary progressive MS. It leads to a steeper accumulation of disability, mostly due to alterations in immune competence, which is demonstrated by both attenuated efficacy of innate and adaptive immune pathways (167). This aged immune system experiences a progressive domination of pro-inflammatory mediators, which transform tissue into a microenvironment favouring sustained inflammation. It eventually facilitates the neurodegeneration culminating in the mosaic pathology intrinsic to MS (168).

In the pathophysiology of MS, the processes of demyelination and neuroinflammation arise from the concerted action of autoreactive T lymphocytes and aberrantly activated microglia (169). Recent investigations have identified the SET domain family of histone methyltransferases as pivotal modulators at the neuro-immune interface (170). SETD5 mediates microglial activation and expression of inflammatory mediators within the CNS (120). SETD2 catalyzes trimethylation of histone H3 at lysine 36, and enhances DNA repair and transcriptional activity within oligodendrocytes; the failure of these processes may result in the acceleration of neurodegenerative processes (46). SETD7 regulates the differentiation of IL-17-producing T helper cells by the methylation and thus ensuing regulation of the transcriptional activity of STAT3 (signal transducer and activator of transcription) (54). Overall, these studies incriminate SETD enzymes as dual-acting regulators in the infiltration of immune cells into the CNS and the ability of neural cells to survive inflammatory offence (32).

### 5.4 Inflammatory bowel disease

IBD (171), comprising Crohn’s disease (172) and ulcerative colitis (173), arises due to genetic, environmental and immune factors when the intestine fails to maintain immune tolerance to resident microbiota in sustaining chronic inflammation (24, 174, 175). The SETD6 mitigates this process by mono-methylating the NF-κB RelA subunit to limit pathogenic NF-κB translocation (24). Loss of SETD6 catalytic function in either the intestinal epithelium or infiltrating leukocytes results in uninterrupted RelA nuclear activity and augmented cytokine production (160). Analogously, diminished SETD1A stability modifies chromatin landscape accessibility, particularly at IL-23 promoter regions, thus amplifying IL-23-Th17 axes that drive intestinal permeability and damage (176).

SETD2 plays fundamental roles in modulating epigenetic mechanisms in connection with histone and DNA modifications, which are crucial for the differentiation and functioning of innate lymphoid cell (ILC3) subpopulations involved in intestinal infections, inflammatory diseases, and tumours (36, 177, 178). SETD2 has also been implicated in the regulation of Treg cells in the intestine for the survival and suppression of colitis (179). SETD8, when downregulated, further compromises epithelial barrier function and abrogates immune checkpoint controls, thus coupling epigenetic defects to IBD chronicity and severity (180). Further, SETDB1 has been demonstrated in the regulation of various processes linked with ageing, tumors and IBD because of methylation of both histone and non-histone proteins (21, 125, 181). Recently, it has been demonstrated that targeting TNF-α, IL-12/IL-23, and JAK-STAT signaling is effective in the clinical management of IBD (182).

Other diseases with immune disorders linked to dysregulated SETD enzymes include psoriasis, atherosclerosis and type 2 diabetes mellitus (183). Altered H3K27 methylation has been observed in keratinocyte proliferation in psoriasis with the involvement of SETD2 and SETD8 (74, 81). It is now strongly documented that epigenetic modifications are strongly linked to cardiovascular diseases, including atherosclerosis (148). SETD8 is related to inflammation-linked vascular calcification through activating Mark4 and Sp1 expression that leads to atherosclerosis (154, 155). SETD7 methyltransferase is also associated with atherosclerosis through the generation of ROS and proinflammatory cytokines (147). However, there are a few reports that SETD7-triggered modification with H3K4me1 methylation and other modifications are linked to type 2 diabetes mellitus (146, 184, 185).

## 6 Mechanisms and consequences of SETD dysfunction

### 6.1 Mutations and aberrant epigenetic alterations

Comprehensive genomic profiling of autoimmune and malignant conditions consistently uncovers recurrent mutations and chromosomal structural rearrangements targeting the SETD gene family members. In autoimmune contexts, truncated or missense mutations that inactivate SETD2 abrogate the deposition of H3K36 mono- and trimethylation. It thus uncouples transcriptional proofreading and inadvertently drives atypical IFN-I gene expression (73). Concurrently, inherited or somatically acquired alterations in SETD1A or SETD1B clone promoter and/or enhancer occupancy reduce H3K4 methylation, dysregulating the transcriptional circuitry of both pro-inflammatory cytokines and immune checkpoint ligands (59). Complementing these genetic perturbations, promoter hypermethylation or competitive inhibition by specific non-coding RNAs silences SETD expression in a subset of thyroid or connective tissue disorders and accentuates the burden of chronic tissue inflammation (**Figure 12**) (32).

### 6.2 Immune cell plasticity and tolerance

The adaptive properties of immune cell lineages, termed plasticity, are shaped by the chromatin landscapes deposited by SETD enzymes (71). SETD1A/B holo-complexes generate H3K4me3 marks that afford promoters and enhancers of IFN-γ, IL-4, and IL-17 transcripts precise levels of transcriptional accessibility, thus guiding naïve CD4^+^ T cells along Th1, Th2, or Th17 fates (69). These findings result in dysregulation of T-cell differentiation toward pathogenic states. SETD7 and SETD6 also regulate immune tolerance. SETD7 levies tertiary suppression on Th17 transcriptional activity (54). SETD8 supports the transcriptional activity of FOXP3^+^ regulatory T cells (162). SETD dysfunction erodes central and peripheral tolerogenic checkpoints. It thereby releases antibody-producing B cells and increases autoreactive T-cells (186). Overall, these studies support the vital role of SETD enzymes in sustained immune equilibrium and restrictive immune-regulated tissue damage (43, 187).

It has been reported that neutrophils show remarkable functional plasticity within chromatin remodeling (183). Citrullination, acetylation and methylation of histones are found crucial in the regulation of neutrophil death (188). Inflammatory signals like TNF-α or LPS trigger explicit histone lysine methylation H3K4me3 and acetylation H3K27ac, resulting in modifications in nucleosomes (189). It thus permits activation of genes involved in antimicrobial and pro-inflammatory features (190). In contrast, cytokine IL-10 is involved in the H3S10 phosphorylation that is linked to the inflammation resolution and renewal of tissue (189). The dysregulation of the enzymes interrupts the equilibrium of neutrophil polarization (191). Further, over functioning of neutrophils will lead to persistent inflammatory conditions like type 2 diabetes, autoimmune disorders, atherosclerosis as chronic inflammatory disorders, including several types of cancer (183). These findings place epigenetically modified neutrophils by the SETD family enzymes at the junction of innate immune plasticity and inflammation (189). It therefore provides a mechanistic relationship between immune dysregulation and chronic inflammatory and oncogenic diseases (191).

## 7 Therapeutic avenues of targeting SETD proteins

### 7.1 Epigenetic and small-molecule inhibitors

**Figure 14:**
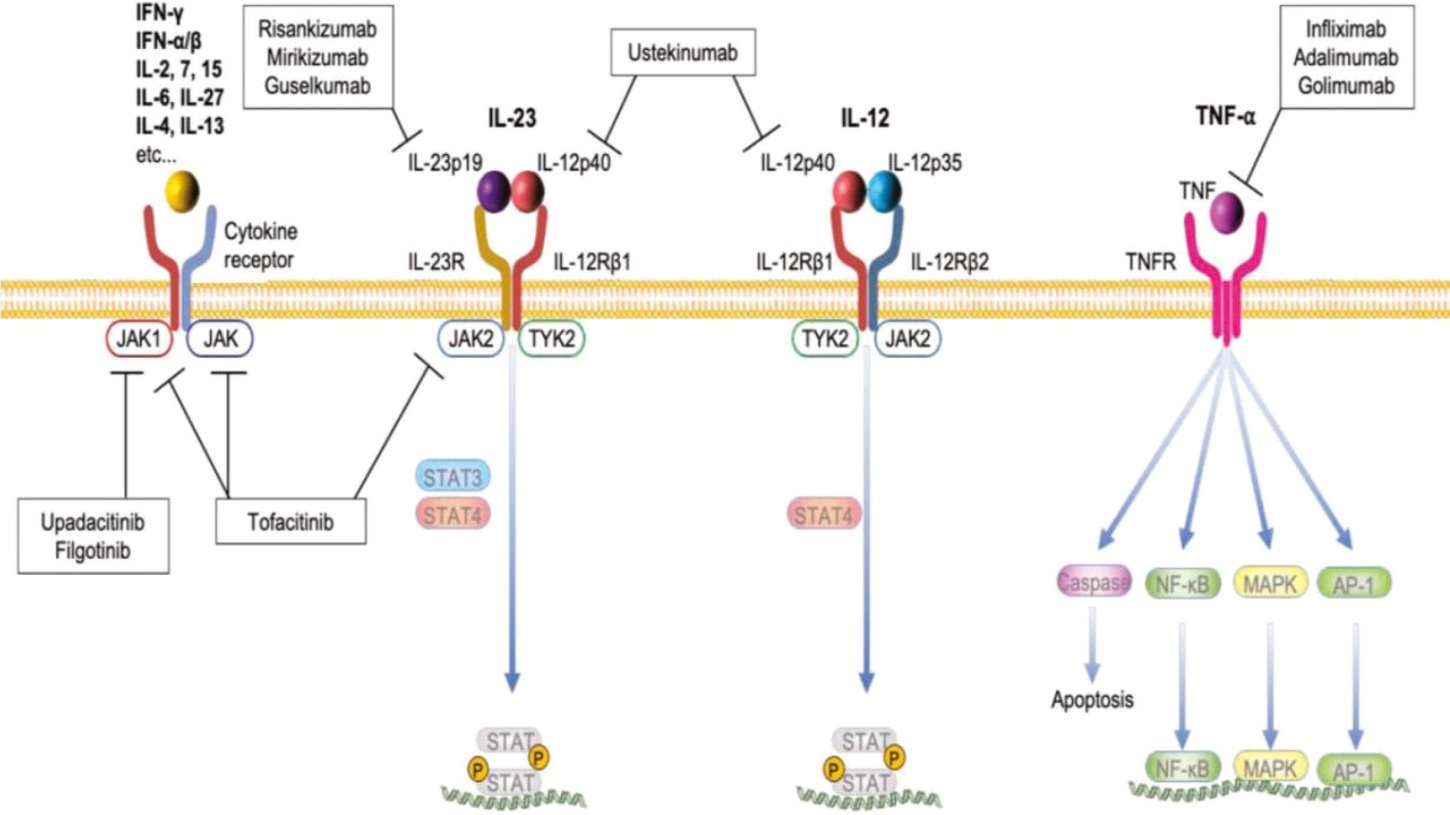
A summary of the signalosomes elicited by the pro-inflammatory cytokines TNF-α, IL-12, and IL-23. These signals converge upon three canonical pathways: NF-κB, mitogen-activated protein kinase (MAPK), and Janus kinase-signal transducer and activator of transcription (JAK-STAT) cascades. Specifically, IL-12 and IL-23, through the heterodimeric receptors, signal in a JAK2-TYK2 axis, catalyzing phosphorylation and subsequent activation of STAT4 and STAT3, in that order; TNF-α predominantly activates both the NF-κB and the p38 and ERK branches of the MAPK pathway. Clinical evidence indicates that therapeutic strategies directed at these cytokines, including antagonists of TNF-α and targeted inhibitors of the JAK-STAT axis, yield detectable and sustained benefits in the management of IBD (182).

Focused programmes are synthesizing small-molecule inhibitors capable of either precisely occluding the catalytic SET domain or of abrogating SETD-substrate docking (192). Inhibitors directed against SETD7, for example, suppress NF-κB and STAT3 signalling circuits and are hence capable of curtailing the production of pro-inflammatory cytokines (54, 193). Concurrently, the pharmacological blockade of SETD8 is being scrutinized for its ability to recalibrate the expression of immune checkpoint surface molecules. This adjustment is expected to amplify anti-tumor immunity in the clinical context (56, 57). Broad-spectrum epigenetic agents, including histone methyltransferase inhibitors, have consistently demonstrated therapeutic benefit in preclinical inflammation-centered models. However, the central obstacle of distinguishing among individual SETD paralogues has yet to be decisively overcome (23, 57). A summary of events of signal transduction and other pro-inflammatory cytokines involved in downstream signaling, including the NF-κB, MAPK, and JAK-STAT pathways, is given below (**Figure 14**) (182).

### 7.2 Epigenome editing and CRISPR-based modulation

The integration of catalytically inactive CRISPR/Cas9 fusions with epigenetic effector domains enables locus-specific perturbation of histone methylation states catalyzed by SETD-family methyltransferases (194). By tethering epigenome remodelers to CRISPR-associated sequence guides, researchers can impose selective and reversible alterations of tri- or di-methylated lysine marks. It will help to reprogram pathogenic transcriptional landscapes to restore immune tolerance (195). Experimental evidence indicates that CRISPR interference directed against the SETD2 or SETD6 loci dampens lineage-typical cytokine production to attenuate T-cell hyperactivity (196). While the application is presently experimental, the data substantiate the concept of programmable elevation or inhibition of SETD activity as a therapeutic axis in immune dysregulation (196).

### 7.3 Translational prospects in precision medicine

Given the therapeutic potential of precision medicine, the challenges associated with the selective silencing of SETD enzymes are rather intimidating (51). A SETD biomarker-driven point mutation strategy associated with tri-methylated histone patterns and tumor cytokine signatures can robustly identify cohorts with SETD-driven disease most likely to benefit from SETD-targeted therapy (33). In cancer, the combination of small-molecule SETD inhibitors with established immune checkpoint blockade may result in additive antitumor effects. Conversely, in autoimmune disease, phenotypic rebalancing of PD-1 and T-regulatory T cell subsets may restore immune self-tolerance (186, 197). The potential union of rational therapeutic design with high-resolution epigenomic features also provides an elegant funnel through which SETD enzymes can shift from mechanistic intrigue to clinically actionable and achievable biomolecular levers (3, 12, 57).

## 8 New paradigms and future prospects

### 8.1 SETD biomarkers for immune dysregulation

SETD enzymes modify the targeted histones and their corresponding transcriptional patterns (46, 122). In SLE, diminished SETD2-dependent H3K36 trimethylation correlates with aberrant IFN-I overdrive, whereas H3K4 trimethylation patterns orchestrated by SETD1A/B correspond with T-cell disproportion in RA (104). It is possible that their transcriptional patterns could serve as biomarkers for immune dysregulation (102). SETD profiling in combination with peripheral histone methylation offers real-time monitoring of disease progression and treatment efficacy. Even so, the ability to study circulating chromatin of peripheral blood boasts the potential to extend the clinical utility of these biomarkers greatly (64, 121).

### 8.2 Synergistic with multi-omic strategies

The epigenome, transcriptome, proteome, and metabolome collected yield a comprehensive framework for investigating the regulatory networks controlled by SETD proteins (43). To date, module-resolved analyses have demonstrated that the SETD6-guided H3K36 tri- and dimethylation suppresses NF-κB-driven transcriptional activation of the inflammatory cascade as well as remodels lipid and phosphate metabolism in activated macrophages (45). Integration of these SETD multi-omics data with other data at the single-cell level provides unprecedented definition of cell lineage-specific SETD patterns dispersed throughout the inflamed joints, gut and airway epithelium. Collectively, these multi-omics strategies could provide a systems-level understanding of cell-cell and micro-environmental relationships that SETD-modulated immune homeostasis (70).

### 8.3 Personalized medicine perceptions

The intrinsic heterogeneity that characterizes autoimmune and inflammatory disorders mandates the adoption of finely calibrated therapeutic approaches (61). In this regard, the SETD family enzymes are positioned centrally since their catalytic profiles are shaped by both germline variances and environmental factors (192). Algorithmic precision treatment models should be formed from SETD germline variations, distributions of chromatin marks, and transcriptional activity of cytokine-regulated genes (64). More targeted anticancer activities with precision medicine may result from the input of immune checkpoint inhibitors and SETD antagonists (57, 69, 198). On the other hand, in autoimmune diseases, SETD modulation reinstates immunological tolerance with a smaller therapeutic window compared with immunosuppressive regimens (197).

### 8.4 Outstanding issues in SETD-immune augmentation

Notwithstanding the significant progress in the field, several challenges still exist (196). A comprehensive study on non-histone substrates needs to be done regarding the resolution and immune homeostasis (70). Detailed investigations into SETD dysfunction and external stimuli in metabolites from the microbiota and infectious agents need to be done (192). The specific design of molecules selective with restricted off-target effects is another challenge (12). It also needs to be ascertained whether biochemical interference can produce sturdy and oppressive effects on immunological memory (8, 197).

## 9 Conclusions

Histone lysine methyltransferases, SETD, are recognized as important epigenetic regulators in immunological investigations (23). They modify histone and non-histone substrates, and change the transcriptional landscapes with immune ancestry differentiation, and augmentation of the volume, pattern, and dynamics of cytokine secretion existence of immune tolerance (33). SETD hypomethylation, mutations, non-canonical SETD methylation patterns and SETD misregulation all trigger the loss of immune homeostasis (70). It results in malfunctioning of the immune system, resulting in a diverse pattern of inflammatory and autoimmune disorders (61). The above review identifies SETD methyltransferases as crucial epigenetic modifiers that amalgamate the dynamics of chromatin remodeling and complicated activities of the immune system (64, 100). New avenues for preclinical and clinical studies may open the therapeutic windows with SETD enzymes as potential biomarkers and therapeutic targets in immune dysregulation using small molecule inhibitors, epigenomic editing (12). The therapeutic control over inflammation within SETD enzymes recalibrates immune responses in autoimmune disorders, avoiding immunosuppression (192). The new small-molecule immune checkpoint inhibitors may work in combination with existing immunotherapies (69). Rapid progress can be made by understanding the molecular biology of SETD, the epigenomics of the patient, and conducting systematic SETD investigations with clinical insights to define a translatable approach for SETD-engineered epigenetic therapies in immune disorders (198).

## Glossary

COMPASS: Complex of proteins associated with SET1
DDR: DNA damage response
Forkhead box P3^+^: FOXP3⁺ regulatory T cells
HATs: Histone acetyltransferases
hnRNPs: Heterogeneous nuclear ribonucleoproteins
HKMTs: Histone lysine methyltransferases
H3K4me3: Trimethylation of histone H3 at lysine 4
IBD: Inflammatory bowel disease
IFN-I: Type I interferon
ISG: Interferon-stimulated genes
KDM1A: Lysine demethylase 1A
MS: Multiple sclerosis
PD-L1: Programmed cell death ligand 1
PTMs: Post-translational modifications
RA: Rheumatoid arthritis
RORγt: Retinoid-related orphan receptor gamma t
SAH: S-adenosyl homocysteine
SAM: S-adenosylmethionine
SET: Su(var)3–9, enhancer-of-zeste, and trithorax
SETD: SET domain–containing
SHI domain: SETD2-hnRNP interaction
SLE: Systemic lupus erythematosus
SMYD2: SET and MYND domain-containing protein 2
SRI domain: SET2-Rpb1 interacting domain
STAT3: Signal transducer and activator of transcription 3
Th17: T helper 17
WW domain: Tryptophan-tryptophan domain
